# TGFβ1 Regulates Human RANKL-Induced Osteoclastogenesis via Suppression of NFATc1 Expression

**DOI:** 10.3390/ijms21030800

**Published:** 2020-01-25

**Authors:** Tadahiro Tokunaga, Sho Mokuda, Hiroki Kohno, Kazutoshi Yukawa, Tatsuomi Kuranobu, Katsuhiro Oi, Yusuke Yoshida, Shintaro Hirata, Eiji Sugiyama

**Affiliations:** Department of Clinical Immunology and Rheumatology, Hiroshima University Hospital, Hiroshima 734-8551, Japan; ta-tokunaga@hiroshima-u.ac.jp (T.T.); sho-mokuda@hiroshima-u.ac.jp (S.M.); hirkohno@hiroshima-u.ac.jp (H.K.); kkyukawa@hiroshima-u.ac.jp (K.Y.); tkurano@hiroshima-u.ac.jp (T.K.); kooi@hiroshima-u.ac.jp (K.O.); fisher37@hiroshima-u.ac.jp (Y.Y.); shirata@hiroshima-u.ac.jp (S.H.)

**Keywords:** human osteoclastogenesis, TGFβ1, RANKL, NFATc1, NF-κB, rheumatoid arthritis

## Abstract

Osteoclasts are multinucleated giant cells responsible for bone resorption. Various mediators involved in osteoclast differentiation have been investigated as possible therapeutic targets for osteoporosis and rheumatoid arthritis (RA). Although transforming growth factor beta1 (TGFβ1) has been described as one such multifunctional cytokine essential for bone remodeling, its effect on osteoclastogenesis remains controversial. Therefore, we sought to examine the effect of TGFβ1 on osteoclast generation induced by receptor activator of nuclear factor (NF)-κB ligand (RANKL) in humans. Peripheral blood monocytes, isolated using magnetic bead sorting, were cultured with macrophage-colony stimulating factor (M-CSF) or RANKL with or without TGFβ1. Tartrate-resistant acid phosphatase (TRAP) staining, as well as bone resorption assays, revealed that TGFβ1 suppressed RANKL-mediated human osteoclast development. Real-time reverse transcription PCR and Western blotting revealed that TGFβ1 reduced the gene and protein expression of nuclear factor of activated T cells, cytoplasmic 1 (NFATc1), the master regulator of osteoclast differentiation, respectively. Luciferase assays indicated that TGFβ1 inhibited the NF-κB p65-stimulated promoter activity of NFATc1. Immunofluorescence analysis demonstrated that TGFβ1 abrogated RANKL-induced nuclear translocation of p65. Thus, TGFβ1 regulates human RANKL-induced osteoclastogenesis via downregulation of NFATc1 by blocking nuclear translocation of NF-κB, suggesting that TGFβ1 may be a potential therapeutic target for RA.

## 1. Introduction

Osteoclasts are multinucleated giant cells with the unique ability of bone resorption [1,2,3,4,5]. Skeletal system homeostasis is maintained via a skeletal metabolic process termed bone remodeling, which involves osteoclast-mediated bone resorption and osteoblast-mediated bone formation [6,7,8,9,10].

Previous studies suggest that osteoclasts differentiate from bone marrow monocyte/macrophage lineage cells in the presence of two indispensable cytokines: macrophage-colony stimulating factor (M-CSF) and receptor activator of nuclear factor (NF)-κB ligand (RANKL) [11,12]. In vitro osteoclast generation following M-CSF and RANKL stimulation of CD14-positive cells (monocytes) isolated from peripheral blood mononuclear cells (PBMCs) has been reported [13]. Osteoclast differentiation in response to M-CSF and RANKL occurs via the formation of perfusion osteoclasts (osteoclast precursor), mature osteoclasts, and resorbing osteoclasts [14]. Transcription factors and related genes are markedly upregulated in the early stages (monocytes and osteoclast precursor). In particular, NFATc1, a master regulator of osteoclast differentiation, induces the expression of downstream osteoclast-specific genes, such as those coding for tartrate-resistant acid phosphatase (TRAP), cathepsin K, calcitonin receptor, and osteoclast-associated receptor (OSCAR) [11]. Cathepsin K is a cysteine protease specific for osteoclasts and plays an important role in degrading the bone matrix proteins during bone resorption [15].

Furthermore, osteoclasts are involved in the pathogenesis of rheumatoid arthritis (RA). RA is an autoimmune disease characterized by systemic synovitis, followed by articular cartilage destruction and bone erosion, which results from increased osteoclast differentiation [2,3,4,5]. Furthermore, treatment with anti-RANKL antibodies (denosumab) strongly suppresses bone erosion in patients with RA [16].

Transforming growth factor beta 1 (TGFβ1) is a cytokine produced by various cells and is involved in multiple biological processes, such as cell differentiation, proliferation, migration, and apoptosis [17,18]. It is the most abundant cytokine (~200 μg/kg) in bones [19] and is one of the crucial factors involved in the communication between bone resorption with bone formation during bone remodeling. In fact, TGFβ1 is one of the ‘classical coupling factors’ that are released after bone absorption and acts to promote osteogenesis [6,7,8,9,10]. However, although TGFβ1 has been described as being critical for bone metabolism, reports on the role of TGFβ1 in osteoclast generation are controversial, and opposing effects of TGFβ1 have been described [20]. For instance, some studies have reported that TGFβ1 stimulates osteoclast development [21,22,23,24,25,26,27,28,29,30,31]. Many of these studies used mouse bone marrow cells or RAW264.7 cells [21,22,23,24,25,26,27,28,29,30], while the others employed human peripheral blood monocytes (PBMs) in the absence of RANKL [31]. Alternatively, other studies have indicated that TGFβ1 inhibits osteoclast differentiation [32,33,34,35]; two such studies used human bone marrow cells [32,33], while the others employed mouse bone marrow cells [34,35]. Other studies have suggested that TGFβ1 demonstrates bidirectional activity [36,37,38,39,40]. Some of these studies demonstrated the bidirectional effects were dose-dependent, with low doses of TGFβ1 promoting osteoclast formation, while high doses had an inhibitory effect [36,37,38]. Further, one study argued a stage-dependency for osteoclast differentiation, indicating that when TGFβ1 is present during only the monocyte stage, human osteoclast generation occurs, whereas continuous TGFβ1 exposure throughout the entire culture period served to inhibit osteoclast development [39]. Finally, a study demonstrated context-dependency when lymphocytes were co-cultured with PBMCs, resulting in enhanced osteoclastogenesis, while PBMC monocultures did not exhibit this effect [40]. Hence, the controversial results surrounding the role of TGFβ1 in osteoclast generation may be due, in part, to the considerable difference in the experimental protocols used in the previous studies.

Therefore, in the present study, we investigated the effect of TGFβ1 on RANKL-induced osteoclast differentiation in human monocytes in a pure-culture system, using magnetic bead-based sorting. We demonstrated that TGFβ1 directly downregulates NFATc1 activity through abrogating nuclear translocation of NF-κB subunit p65, thereby inhibiting human osteoclastogenesis and bone resorption. Our findings provide new insights into the mechanisms underlying the effect of TGFβ1 on human osteoclast generation and into novel therapeutic targets for RA.

## 2. Results

### 2.1. TGFβ1 Inhibits RANKL-Induced Osteoclastogenesis in Human Peripheral Blood Monocytes (PBMs)

**Figure 1:**
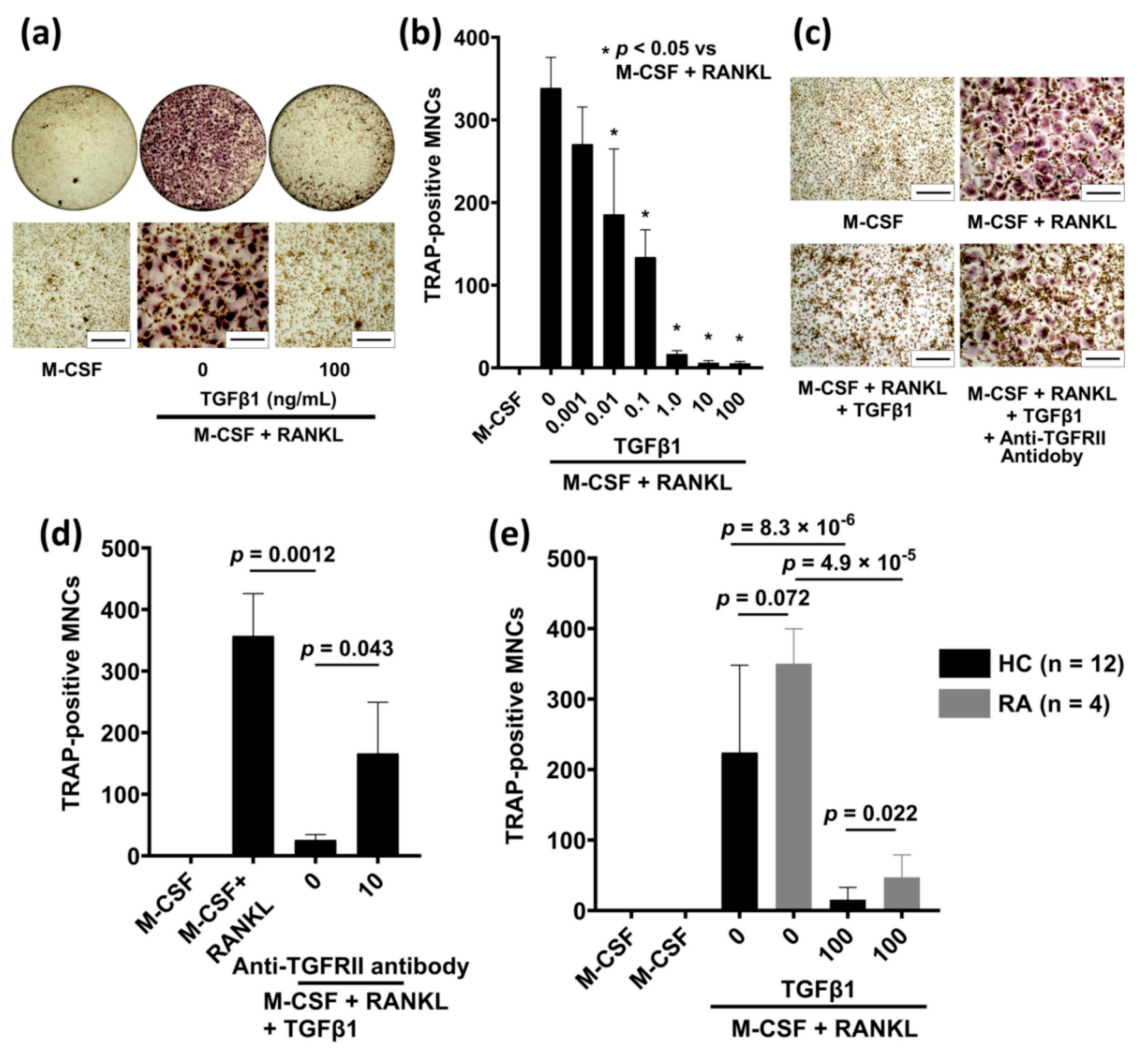
Inhibitory effect of TGFβ1 on receptor activator of nuclear factor kappa-B ligand (RANKL)-induced osteoclastogenesis in human peripheral blood monocytes (PBMs). (**a**,**b**) PBMs were cultured with 50 ng/mL of macrophage-colony stimulating factor (M-CSF) and/or 100 ng/mL of RANKL in the presence of increasing concentrations (0–100 ng/mL) of TGFβ1, for 5–6 days, and then analyzed, using tartrate-resistant acid phosphatase (TRAP) staining. TGFβ1 significantly inhibited RANKL-induced osteoclast generation even at a low concentration of 10 pg/mL, and this inhibitory effect increased in a dose-dependent manner. The inhibitory effect of TGFβ1 reached a plateau at concentrations of ≥1.0 ng/mL. * *p* < 0·05 versus M-CSF + RANKL. (**c**,**d**) TRAP staining-based analysis of the effect of anti-TGFβ receptor II (TGFBRII) antibody on the inhibitory effect of TGFβ1 on RANKL-mediated osteoclastogenesis in human PBMs. PBMs were pretreated with or without 10 μg/mL of TGFBRII antibody for 5 min and then subjected to M-CSF and RANKL treatment in the presence or absence of TGFβ1. Anti-TGFBRII antibody significantly reversed the inhibitory effect of TGFβ1. (**e**) Comparison of the inhibitory effect of TGFβ1 on RANKL-stimulated osteoclastogenesis in PBMs obtained from healthy donors and untreated seropositive patients with rheumatoid arthritis (RA). During the culture, half the culture medium was replaced with fresh medium containing cytokines every three days. Representative microphotographs are shown. Scale bar = 50 μm. Data are presented as the mean ± standard deviation.

First, we examined the effect of TGFβ1 on RANKL-induced osteoclastogenesis in human PBMs. Here, PBMs were purified from PBMCs and consisted of CD14-positive cells (>92%), as determined by flow cytometric analysis. The number of TRAP-positive multinucleated cells (MNCs) in the PBMs cultured with both M-CSF and RANKL was considerably higher than that in the PBMs cultured with M-CSF alone (Figure 1a,b). However, the number of TRAP-positive MNCs in PBMs cultured with M-CSF, RANKL, and TGFβ1 was considerably lower than that in PBMs cultured with M-CSF and RANKL. These data show that RANKL treatment induced osteoclast generation from PBMs and that TGFβ1 potently inhibited this RANKL-induced osteoclastogenesis. Moreover, the suppressive function of TGFβ1 increased in a dose-dependent manner (0–100 ng/mL) and reached a plateau at concentrations ≥ 1.0 ng/mL (Figure 1b). Significant inhibition of osteoclastogenesis was observed at TGFβ1 concentration as low as 10 pg/mL. Moreover, cytotoxicity assay demonstrated that TGFβ1 does not appear to be cytotoxic to human PBMs, and BrdU assay results revealed that TGFβ1 stimulated cell proliferation during osteoclastogenesis, suggesting that the inhibitory effect of TGFβ1 on osteoclast differentiation was not due to cytotoxicity or inhibition of cell proliferation (Figures S1 and S2).

### 2.2. Anti-TGFBRII Antibody Blocks the Inhibitory Effect of TGFβ1 on RANKL-Induced Osteoclastogenesis

TGFβ1 functions via binding to its receptor, TGFβ receptor II (TGFBRII), present on the surface of cells [20,41]; therefore, to determine whether TGFβ1 functions through TGFBRII, we evaluated the effect of anti-TGFBRII antibody on the inhibitory effect of TGFβ1 on RANKL-induced osteoclast differentiation, using TRAP staining. Here, the PBMs were pretreated with anti-TGFBRII antibodies (10 μg/mL) and incubated with M-CSF, RANKL, and TGFβ1 (1.0 ng/mL); the generated osteoclasts were identified with TRAP staining. RANKL treatment induced osteoclast generation from PBMs, and TGFβ1 potently inhibited such osteoclastogenesis; while TGFβ1-induced inhibition of osteoclastogenesis was significantly reduced in PBMs pretreated with anti-TGFBRII antibody (Figure 1c,d). These data confirm the TGFBRII-mediated inhibitory effect of TGFβ1 on RANKL-induced osteoclastogenesis.

### 2.3. TGFβ1 Treatment Reduces RANKL-Induced Osteoclastogenesis in Patients with RA

Osteoclasts are important players during bone erosion occurring in RA. Therefore, we compared the inhibitory effect of TGFβ1 on RANKL-induced osteoclastogenesis in PBMs from patients with RA (*n* = 4) to those from healthy donors (*n* = 12). TGFβ1 significantly inhibited RANKL-induced osteoclastogenesis in PBMs from both healthy controls and patients with RA (Figure 1e). Notably, RANKL-induced osteoclast generation in PBMs from patients with RA was higher than that in PBMs from healthy controls. Moreover, the number of TRAP-positive MNCs generated from PBMs treated with M-CSF, RANKL and TGFβ1 in healthy controls were significantly lower than those in patients with RA (*p* = 0.022, Figure 1e). Our data implies that PBMs in patients with RA exhibit the potential to generate osteoclasts excessively and that the inhibitory response of TGFβ1 in patients with RA is lower than that in controls.

### 2.4. Time-Dependent Effect of TGFβ1 on RANKL-Induced Osteoclastogenesis in Human PBMs

**Figure 2:**
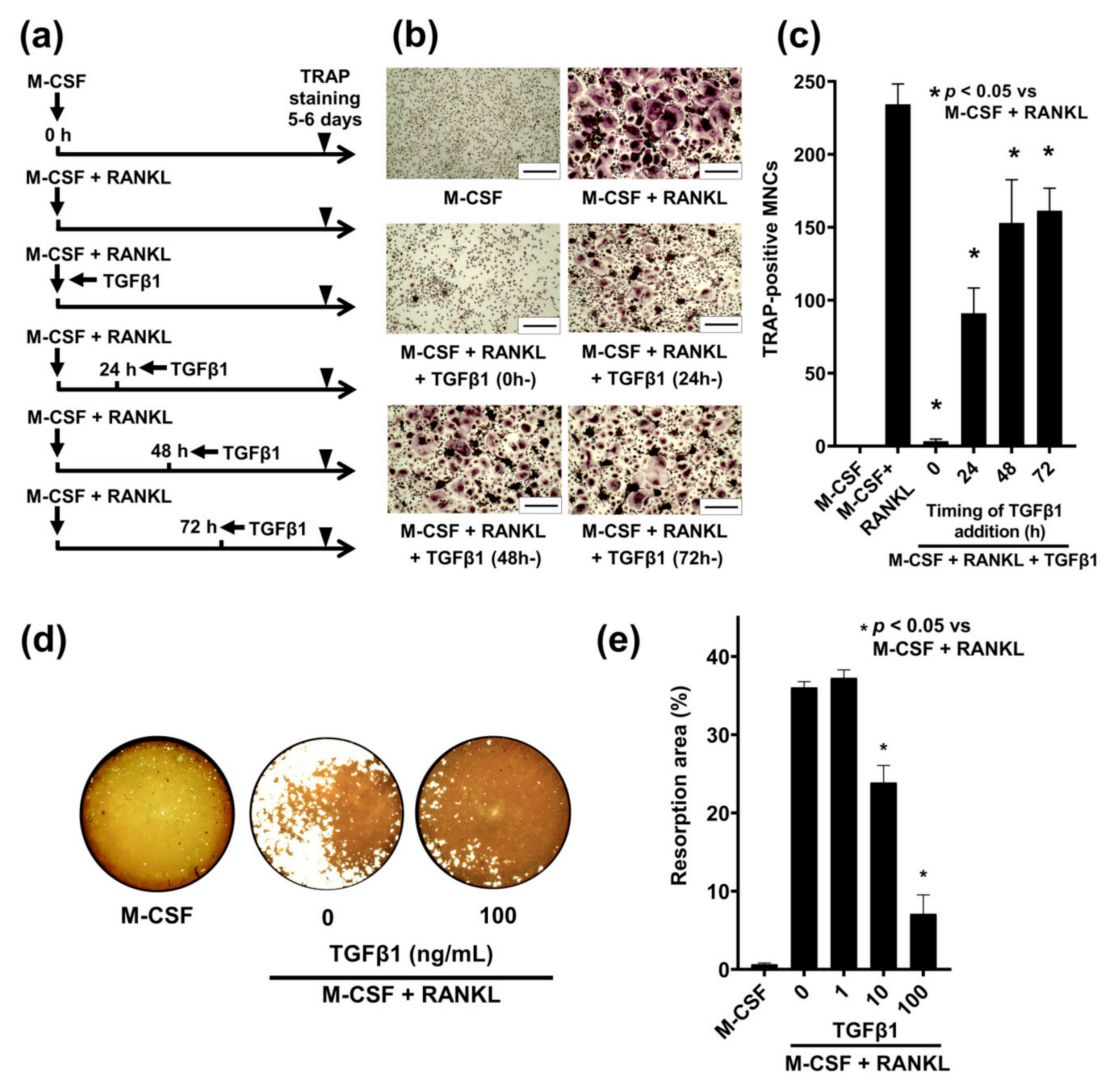
Time-dependent inhibition of osteoclastogenesis and inhibition of bone resorption by TGFβ1 in humans. (**a**) TGFβ1 (100 ng/mL) was added to the peripheral blood monocyte (PBM) culture system at 0, 24, 48, and 72 h after baseline. (**b,c**) The cells were analyzed by tartrate-resistant acid phosphatase (TRAP) staining. The number of TRAP-positive multinucleated cell (MNCs) at the timing of TGFβ1 addition was subtracted from those at the end of the culture period, to normalize the number of MNCs and evaluate the net effect of TGFβ1 on the number of MNCs. Addition of TGFβ1 at 0 and 24 h potently inhibited RANKL-induced osteoclastogenesis. (**d**) Bone resorption activity was analyzed, using Osteo assay plates. PBMs were cultured with M-CSF (50 ng/mL) and RANKL (100 ng/mL) with or without TGFβ1 (100 ng/mL) on Osteo assay surface 96-well plates for 7–12 days, followed by cell lysis and silver nitrate staining. (**e**) TGFβ1 inhibited RANKL-mediated bone resorption activity in a dose-dependent manner. TGFβ1 at concentration ≥10 ng/mL significantly suppressed the RANKL-mediated bone resorption activity. * *p* < 0.05 versus M-CSF + RANKL. During the culture, half the culture medium was replaced with fresh medium containing cytokines and TGFβ1 every three days. Representative microphotographs are shown. Scale bar = 50 μm. The data are presented as the mean ± standard deviation.

Next, we investigated the time dependency of the inhibitory effect of TGFβ1 on RANKL-induced osteoclastogenesis in human PBMs. We treated PBMs with M-CSF (50 ng/mL) and RANKL (100 ng/mL) with or without TGFβ1 (100 ng/mL) stimulation, which was initiated at four different time points (0, 24, 48, and 72 h after baseline) and continued to the end of incubation. The generated osteoclasts were identified using TRAP staining after 5–6 days (Figure 2a). We then subtracted the number of TRAP-positive MNCs at the timing of TGFβ1 addition from those at the end of the culturing period, to normalize the number of MNCs and evaluate the net effect of TGFβ1 on the number of MNCs. TGFβ1 treatments initiating at 0 and 24 h after baseline potently reduced osteoclast generation (Figure 2b,c). These data not only show that TGFβ1 mainly inhibited the early stage of RANKL-mediated osteoclast differentiation but also indicate that TGFβ1 might inhibit after the middle stage of that.

### 2.5. TGFβ1 Inhibits RANKL-Mediated Bone Resorption Activity

Next, we studied the inhibitory effect of TGFβ1 on RANKL-induced bone resorption activity by using Osteo assay plates. RANKL treatment induced bone resorption activity of PBMs, whereas TGFβ1 potently inhibited this RANKL-mediated bone resorption (resorption areas were 36.0% versus 7.1%, respectively; *p* = 3.9 × 10^−5^; Figure 2d,e) in a dose-dependent manner (Figure 2e). These results are consistent with the TRAP-staining results described in Section 2.1. To further verify the effect of TGFβ1 on osteoclast activity, we examined its effect on mature osteoclasts and found that TGFβ1 suppresses mature osteoclast activity, as well as osteoclast differentiation (Figure S3).

### 2.6. Stage-Dependent Differentiation Effect of TGFβ1 on RANKL-Induced Osteoclastogenesis in Human PBMs

Using TRAP staining and bone resorption assays, we also examined the effect of TGFβ1 during the different stages of differentiation on human osteoclastogenesis. Results show that, when TGFβ1 was present only during the monocyte stage, suppression of RANKL-induced osteoclast differentiation was observed in human PBMs (Figure S4). These data support our results that TGFβ1 primarily inhibits the early stage of RANKL-regulated osteoclastogenesis.

### 2.7. TGFβ1 Suppresses the Cell Surface Expression of CD14 and CD11b in Human Osteoclast Precursors

Monocytes and osteoclast precursors express CD14 and CD11b on their cell surface, and both CD14-positive and CD11b-positive monocytes are considered as osteoclast progenitors [42,43]. Furthermore, TGFβ1 was reported to suppress CD14 and CD11b expression in human PBMs [44,45].

**Figure 3:**
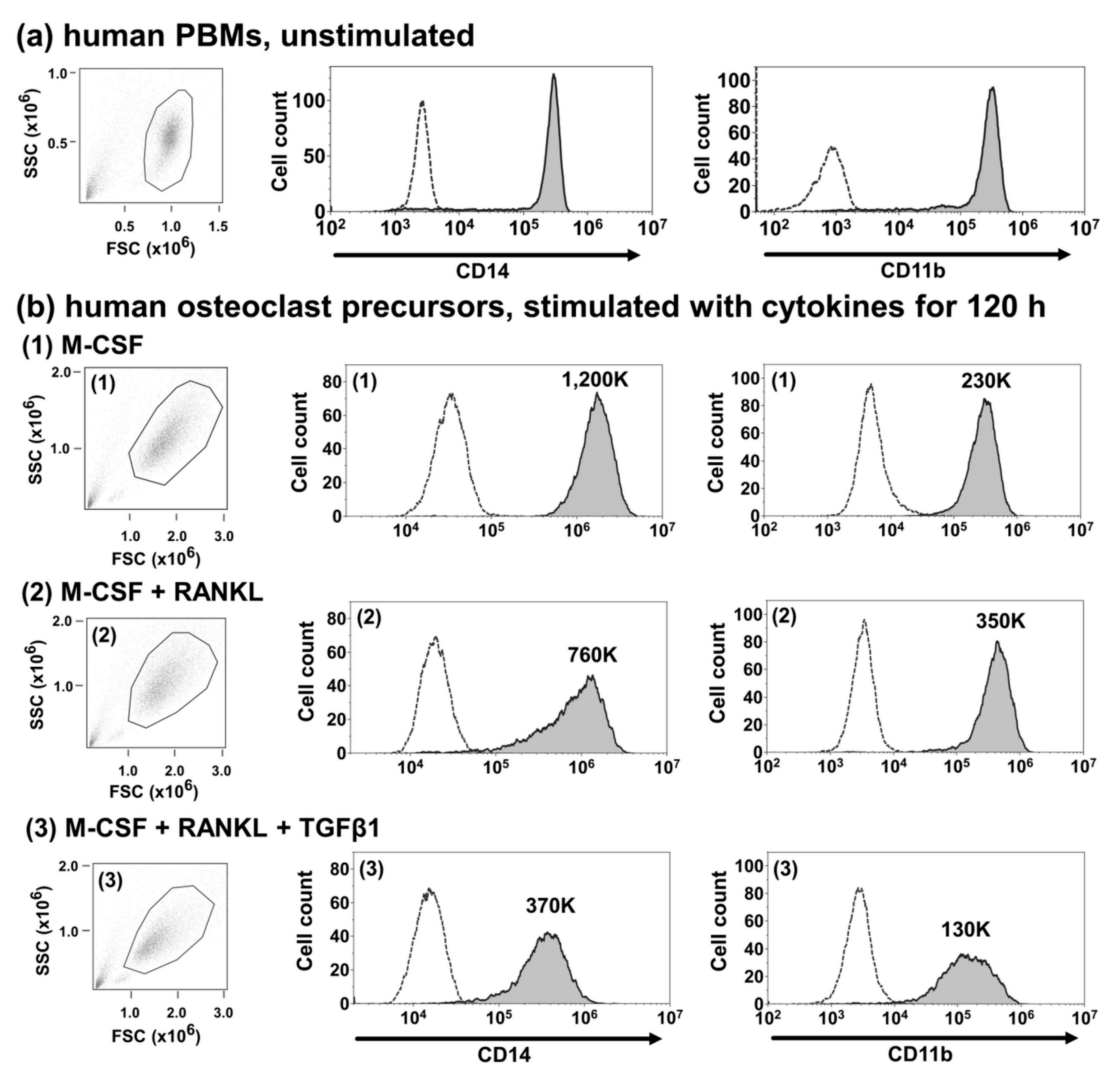
Inhibitory effect of TGFβ1 on the cell surface expression of CD14 and CD11b of human peripheral blood monocytes (PBMs) and osteoclast precursors. (**a**) Human PBMs were freshly prepared from healthy donors. The monocyte populations were gated in a cytogram. (**b**) Human PBMs were cultured with (**1**) M-CSF (50 ng/mL) alone, (**2**) M-CSF and RANKL (100 ng/mL), or (**3**) M-CSF, RANKL, and TGFβ1 (100 ng/mL) for 120 h, and plastic-dish-adherent cells were harvested as osteoclast precursors, using Accutase treatment. The cell populations considered as osteoclast precursors within the same areas were gated in each cytogram. Cells were stained with fluorescein isothiocyanate (FITC)-conjugated anti-human CD14 antibodies (left shaped histogram) and APC-conjugated anti-human CD11b antibodies (right shaped histogram). FITC- and APC-conjugated mouse IgG (left and right unshaped histogram, respectively) were similarly incubated as isotype controls. The cytogram horizontal and vertical axes show forward scatter (FSC) and side scatter (SSC), respectively. A representative result of three independent experiments from different healthy donors is shown. The numbers on each histogram indicate the net mean fluorescence intensity (MFI) calculated as the difference between the MFIs of the shaped and unshaped histograms.

Therefore, we evaluated the effect of TGFβ1 on the cell surface expression of CD14 and CD11b in human PBMs and osteoclast precursors from three healthy donors. Representative histographs for freshly prepared PBMs and osteoclast precursors (PBMs cultured with M-CSF alone and M-CSF and RANKL with or without TGFβ1 for 120 h) are shown in Figure 3a,b, respectively. We determined the net mean fluorescence intensity (MFI), which is the difference between the MFI of histogram of antigen-specific antibodies and the MFI of isotype controls. The net MFIs of both CD14 and CD11b of osteoclast precursors cultured with M-CSF, RANKL, and TGFβ1 (CD14: 370K, CD11b: 130K) were significantly lower than those of osteoclast precursors cultured with M-CSF and RANKL (CD14: 760K, CD11b: 350K; CD14: *p* = 0.026, CD11b: *p* = 0.015). These data suggest that TGFβ1 inhibited osteoclastogenesis via suppressing the cell surface expression of CD14 and CD11b.

### 2.8. TGFβ1 Suppresses the Gene and Protein Expression of NFATc1 and Cathepsin K

**Figure 4:**
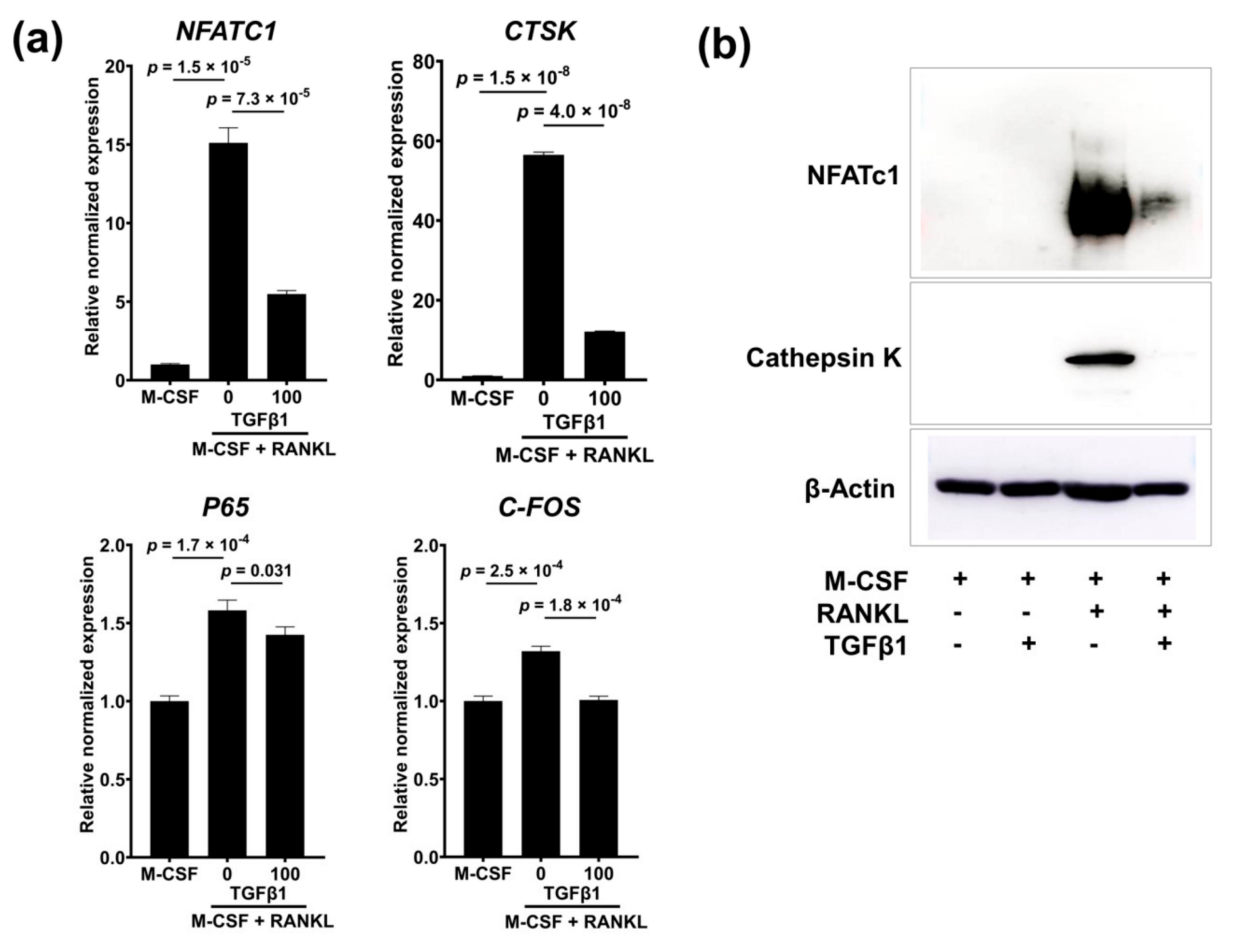
Inhibitory effect of TGFβ1 on RANKL-induced expression of nuclear factor of activated T cells, cytoplasmic 1 (NFATc1) and cathepsin K in human peripheral blood monocytes (PBMs). PBMs were cultured at a cell density of 2.0 × 10^5^ cells/well in a 24-well culture plate, in the presence of M-CSF (50 ng/mL) and RANKL (100 ng/mL), with and without TGFβ1 (100 ng/mL), for 1–5 days. (**a**) Gene expression levels of *NFATC1*, *CTSK*, *P65*, and *C-FOS* were measured by real-time reverse-transcription polymerase chain reaction (RT-qPCR). Total RNA from PBMs cultured for 24 h was harvested for p65 and c-Fos. Total RNA from PBMs cultured for 48 and 96 h was harvested for NFATc1 and cathepsin K, respectively. The data were normalized to *ACTB* expression and are presented as the mean ± standard deviation (*n* = 3). (**b**) Protein levels of NFATc1 and cathepsin K were determined by Western blotting. Proteins were harvested from PBMs cultured for 120 h. A representative photograph of three independent experiments from different healthy donors is shown.

To further elucidate the mechanism underlying the observed TGFβ1-induced inhibition of osteoclastogenesis, we examined the effect of TGFβ1 on RANKL-induced gene and protein expression of the critical osteoclast-related signaling proteins, NFATc1 and cathepsin K. Real-time reverse-transcription polymerase chain reaction (RT-qPCR) and Western blotting showed that RANKL treatment increased *NFATC1* and *CTSK* gene expression (Figure 4a) and the respective protein levels (Figure 4b), and TGFβ1 treatment suppressed this RANKL-induced increase in expression. Thus, TGFβ1 regulated RANKL-induced expression of NFATc1 and its downstream targets, such as cathepsin K.

We next examined the effect of TGFβ1 on RANKL-induced gene expression of two transcription factors, namely NF-κB subunit p65 and c-Fos. RT-qPCR analysis demonstrated that RANKL treatment slightly enhanced *P65* and *C-FOS* gene expression, while TGFβ1 treatment suppressed them (Figure 4a). However, the inhibitory effect of TGFβ1 on *P65* and *C-FOS* gene expression was modest compared with that of *NFATC1* and *CTSK.*

### 2.9. TGFβ1 Directly Inhibits NFATc1 Promoter Activity

**Figure 5:**
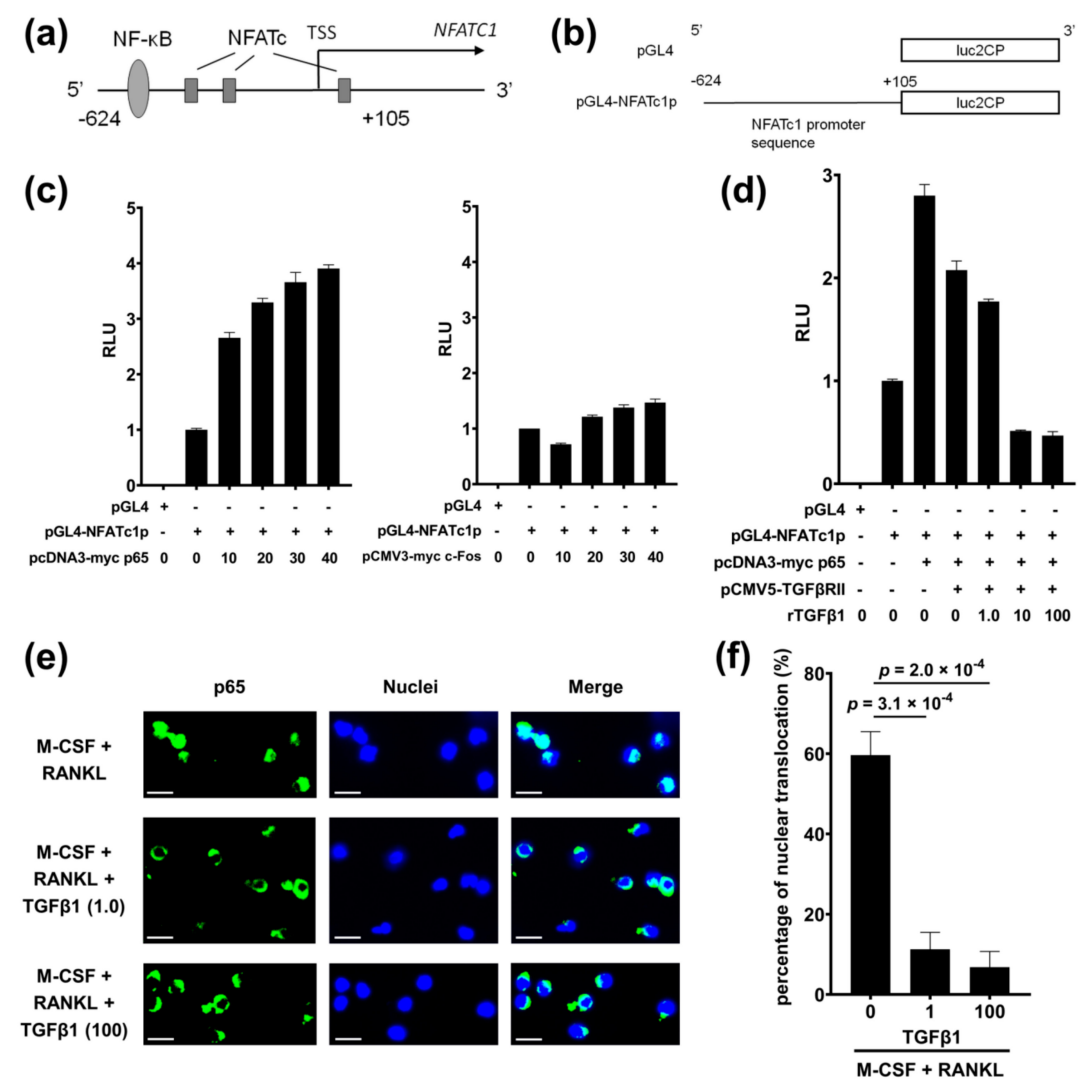
Inhibitory effect of TGFβ1 on NFATc1 promoter activity and p65 nuclear translocation induced by RANKL. (**a**) Schematic drawing of NFATc and NF-κB-binding motifs on the −624/+105 regions of the human *NFATC1* gene. We identified the binding motif sequences (score > 10 points) on these regions, using the JASPAR database (jaspar2014.gnereg.net). (**b**) Schematic drawing of pGL4-basic plasmid (upper) and a luciferase (luc2CP)-linked NFATc1 promoter sequence containing pGL4 plasmid (pGL4-NFATc1p) (bottom). (**c**) Human embryonic kidney (HEK) 293T cells (1.0 × 10^5^ cells/well in a 12-well plate) were transfected with the indicated plasmids (pGL4-basic plasmid, 200 ng; reporter plasmid (pGL4-NFATc1p), 200 ng; pcDNA3-myc p65, 0–40 ng; pCMV3-myc c-Fos, 0–40 ng; and mock plasmid (pcDNA3 vector)). (**d**) HEK293T cells were transfected with the indicated plasmids (pGL4-basic plasmid, 200 ng; reporter plasmid (pGL4-NFATc1p), 200 ng; pcDNA3-myc p65, 40 ng; pCMV5-TGFβRII, 100 ng; and mock plasmid (pcDNA3 vector)). After transfection, recombinant TGFβ1 (rTGFβ1; 0–100 ng/mL) were added to the culture for 4–6 h prior to lysis. (**e**,**f**) Immunofluorescence analysis and quantitative analysis of p65 nuclear translocation induced by RANKL stimulation. TGFβ1 significantly abrogated RANKL-induced nuclear translocation of p65 in a dose-dependent manner. Representative microphotographs are shown. Scale bar = 20 μm. The data are presented as the mean ± standard deviation.

NF-κB is a downstream transcription factor of the RANKL–RANK signaling pathway and is structurally comprised of several subunits such as p50, p52, and p65 [46,47]. NF-κB is important for the initial induction of NFATc1 [11,12]. In fact, an NF-κB-binding motif has been identified within the human NFATc1 promoter region, using the JASTAR database (jaspar2014.gnereg.net) (Figure 5a). c-Fos is also a downstream transcription factor in the RANKL–RANK signaling pathway and a critical component of the transcription factor complex activation protein (AP)-1 [11,12], which cooperates with the robust induction of NFATc1 through autoamplification of NFATc1 [48]. Therefore, to evaluate the direct effect of TGFβ1 on NFATc1 promoter activity, we performed luciferase assays by using a reporter plasmid (pGL4-NFATc1p) containing the human *NFATC1* gene promoter sequence (Figure 5b) and human embryonic kidney (HEK) 293T cells. Endogenous *NFATC1* expression levels in HEK293T cells were extremely low compared to those in PBMs (Figure S5a). First, we examined the effect of p65 and c-Fos on the NFATc1 promoter region and found that overexpressed p65 enhanced luciferase activity in a dose-dependent manner, whereas overexpressed c-Fos induced minimal levels of luciferase activity (Figure 5c) (Figure S5b). Next, to evaluate the effect of TGFβ1 on p65-mediated NFATc1 promoter activity, pCMV5-TGFβRII plasmid was also transfected, and subsequently recombinant TGFβ1 were added. TGFβ1 inhibited the luciferase activity in a dose-dependent manner (Figure 5d and Figure S5c). These data show that p65 functioned as a transcription factor and enhanced NFATc1 promoter activity, and that TGFβ1 directly inhibited this p65-induced enhancement of NFATc1 promoter activity.

### 2.10. TGFβ1 Inhibits Nuclear Translocation of p65 Induced by RANKL

To elucidate the mechanism employed by TGFβ1 to downregulate p65-stimulated NFATc1 promoter activity, we investigated the effect of TGFβ1 on RANKL-induced nuclear translocation of p65 via immunofluorescence assay. TGFβ1 suppressed p65 nuclear translocation induced by RANKL in a dose-dependent manner (Figure 5e,f), indicating that TGFβ1 downregulates p65-induced NFATc1 activity by blocking nuclear translocation of p65.

## 3. Discussion

TGFβ1 is produced by various cells and is involved in multiple biological processes, such as cell differentiation, proliferation, migration, and apoptosis [17,18]. TGFβ1 is critical for skeletal metabolism, including bone remodeling. However, previous reports on the effects of TGFβ1 on osteoclast generation are contradictory, partially because of the differences in the experimental systems, such as cell species, single-population culture, or cell co-culture. It has been demonstrated that stromal cells or lymphocytes could induce osteoclast development in the presence of monocytes [49,50]. In addition, most studies investigated murine osteoclast generation, and only few studies used human cells; it was reported that certain molecules, such as prostaglandin E2 (PGE2), exert opposing effects in murine and human osteoclast generation [51]. Therefore, in the present study, we investigated the direct effect of TGFβ1 on RANKL-mediated osteoclast generation in human osteoclast system, based on monocytes that were purified by using magnetic bead sorting. In the present study, both TRAP staining and bone resorption assays revealed that TGFβ1 inhibited osteoclastogenesis in PBMs in a dose-dependent manner.

We also evaluated osteoclastogenesis in PBMs from patients with RA and healthy controls. Relatively increased osteoclast generation was observed after RANKL treatment of PBMs from untreated seropositive patients with RA (positive for anti-cyclic citrullinated peptide antibody), compared to the healthy controls. These results are consistent with the previous reports stating higher osteoclastogenesis in PBMs from patients with RA [52,53]. However, the response to TGFβ1 differs between these two populations; the number of TRAP-positive MNCs generated from PBMs treated with M-CSF, RANKL, and TGFβ1 in healthy controls was significantly lower than those in patients with RA. This finding indicates a difficulty in suppression of osteoclastogenesis from PBMs in patients with RA, compared with that in healthy controls. Although we examined the TGFβ1-mediated inhibition of RANKL-induced osteoclastogenesis in RA patients compared with that in healthy controls, the number of samples was very small. Therefore, to verify these findings, further studies with larger sample sizes should be conducted. This discrepancy in response to TGFβ1 might be implicated in the pathogenesis of RA; however, the reason for this difference remains unclear in the present study.

To the best of our knowledge, only two studies have evaluated the effect of TGFβ1 on osteoclast differentiation from human PBMs stimulated by RANKL, using magnetic bead sorting [39,40]. One concluded that TGFβ1 promoted human osteoclastogenesis in PBMs via stimulation of p38 mitogen-activated protein kinase (MAPK), whereas continuous exposure to TGFβ1 inhibited osteoclastogenesis via downregulation of RANK expression and the subsequent suppression of RANKL-RANK signaling [39]. The other concluded that TGFβ1 enhanced osteoclastogenesis in PBMCs in the presence of lymphocytes but not in purified CD14-positive monocytes. Thus, the study indicated that the effect of TGFβ1 was context-dependent [40]. Moreover, our results show that TGFβ1 strongly suppressed the early stage of RANKL-induced osteoclast differentiation. The reason for this discrepancy between our results and those from these previous studies is unclear; however, it may be partly because of the differences in experimental protocols and culture conditions, such as cellular density and reagent concentration.

CD14 and CD11b play important roles in osteoclastogenesis. CD14 is known to be the binding site on the monocyte lineage cells for lipopolysaccharide (LPS) and LPS-binding protein (LBP) complex [44]. Several previous studies demonstrated that osteoclasts were differentiated exclusively from CD14+ monocytes and suggested that CD14+ monocytes were the primary source of osteoclast precursors [13,14,52,54,55]. Additionally, a recent study indicated that relatively high expression of Runx2, an essential transcription factor for osteoclast differentiation, in CD14+CD16- monocytes may explain its enhanced osteoclastogenesis [56]; however, another study showed that CD14- monocytes in bone slices neither formed osteoclasts nor demonstrated any resorbing activity [55]. Alternatively, higher proportions of CD14+ osteoclast precursors were reported to contribute to the enhanced osteoclastogenesis in PBMs from patients with RA [53]. TGFβ1 was reported to suppress CD14 expression on human PBMs [44]. CD11b interacts with CD18 to form the CD11b/CD18 integrin pair (macrophage antigen 1, Mac-1). Blocking CD11b was shown to inhibit RANKL-mediated osteoclast differentiation, suggesting its importance in osteoclastogenesis [57]. Moreover, the same group demonstrated that the interaction of CD11b/CD18 with its counter receptor, intracellular adhesion molecule (ICAM)-2, may serve as a trigger for osteoclast differentiation and, therefore, play an important role in osteoclastogenesis [58]. Another study revealed that CD11b promoted RANKL-induced osteoclastogenesis through the spleen tyrosine kinase (Syk) signaling pathway [59]. CD11b is considered to be an important marker of osteoclast precursor in humans [42], and TGFβ1 was reported to suppress CD11b expression on human PBMs [45]. In our study, we evaluated the effect of TGFβ1 on the cell surface expression of CD14 and CD11b in human osteoclast precursors, and we found that TGFβ1 inhibited the expression of both CD14 and CD11b. Collectively, these findings indicate that downregulation of CD14 and CD11b by TGFβ1, as well as a skewed distribution of osteoclast precursors toward CD14- and CD11b- cells, may contribute to the inhibition on RANKL-mediated osteoclastogenesis. Although our experimental protocols differed markedly from those of previous studies that showed suppression of CD14 and CD11b expression by TGFβ1 without using M-CSF and RANKL, our results are consistent with those of these studies [44,45].

RANK–RANKL signaling is essential for osteoclast differentiation; RANKL binding to its receptor RANK triggers adaptor molecules such as tumor necrosis factor (TNF) receptor-associated factor (TRAF) 6 to activate two transcription factors, NF-κB and c-Fos, which are important for induction of NFATc1. Subsequently, NFATc1, a master regulator of osteoclast differentiation, is activated by calcium oscillation signaling and binds to its own promoter, thereby causing autoamplification. Finally, NFATc1 activates osteoclast-specific genes, such as TRAP and cathepsin K, in coordination with other transcription partners, such as NF-κB, AP-1 complex, and PU.1 [11]. A previous study showed that both p50 and p52 NF-κB subunits regulated the first stage of murine osteoclast generation, followed by c-Fos and NFATc1 [48]. Another study revealed that the p65 NF-κB subunit enhanced mouse NFATc1 promoter activity within 24 h after RANKL stimulation, indicating that NF-κB is important for the initial induction of NFATc1 [60]. We then used reporter assays to confirm the direct involvement of TGFβ1 in the expression of NFATc1 and found that p65-enhanced NFATc1 promoter activity in humans (Figure 5c). In addition, we showed that the p65-mediated NFATc1 promoter activity was directly suppressed by TGFβ1. To further elucidate the mechanism, we also demonstrated that TGFβ1 downregulated NFATc1 activity by abrogating nuclear translocation of p65. However, we were unable to detect the relationship between c-Fos and NFATc1 promoter activity on the −624/+105 regions located around the transcription start site (TSS) in the human *NFATC1* gene. We did not identify any regions containing the c-Fos (AP-1) binding motif, using the JASTAR database (Figure 5a). However, considering that previous studies described c-Fos involvement in mouse Nfatc1 transcription activity [48,60], c-Fos may function on other human promoter regions outside the −624/+105 regions, which we investigated in this study, or on the mouse Nfatc1 promoter region.

**Scheme 1:**
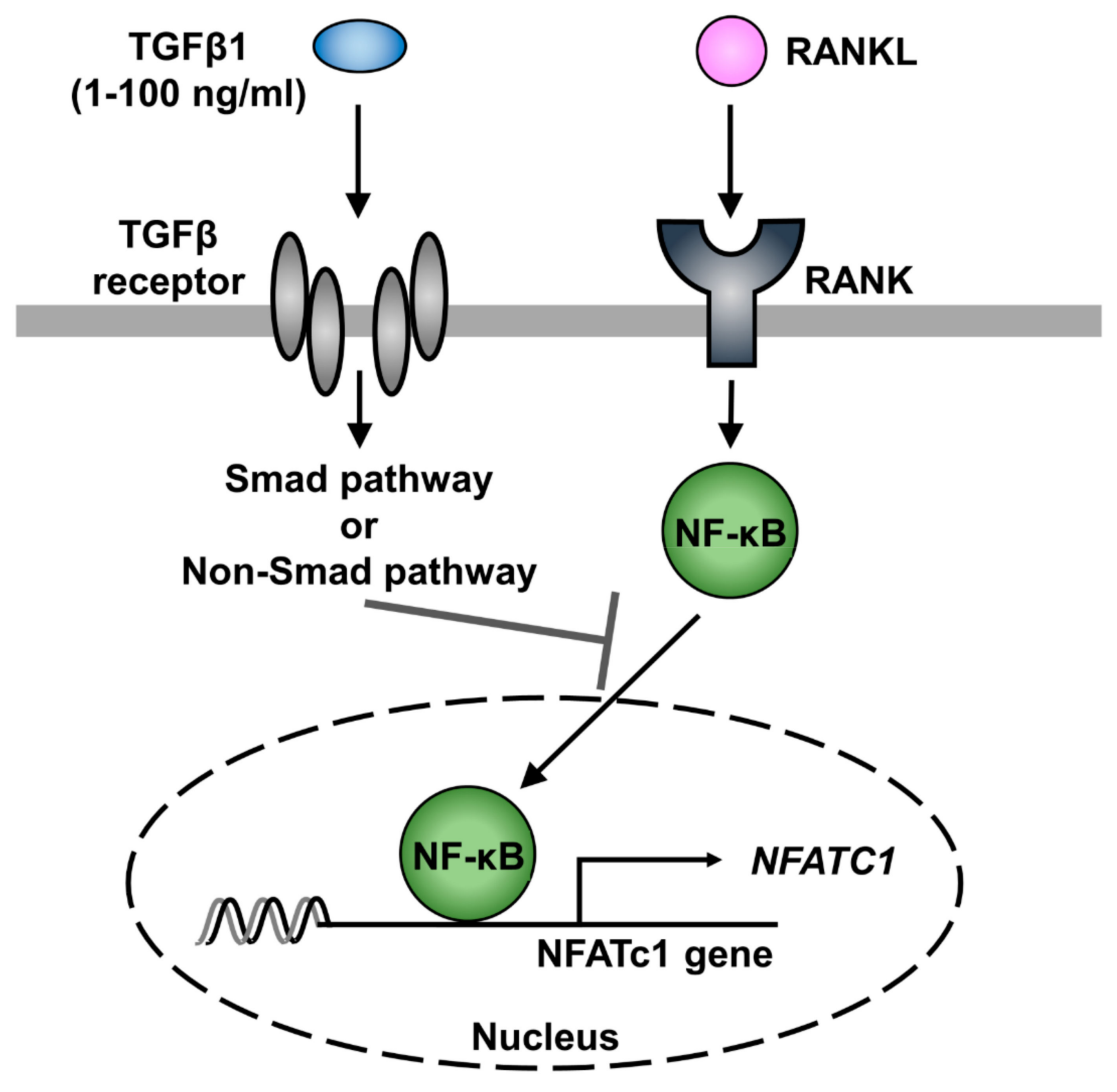
Schematic drawing of the role of TGFβ1 in the regulation of NFATc1. We propose that NF-κB (p65) induced by RANKL stimulation enhances the expression of *NFATC1* gene as a master regulator of osteoclastogenesis and that TGFβ1 (1–100 ng/mL) inhibits NFATc1 promoter activity by blocking nuclear translocation of p65.

In summary, our data indicate that TGFβ1 suppresses RANKL-induced osteoclast development via downregulation of NFATc1 expression, by blocking nuclear translocation of p65 (Scheme 1). These findings are consistent with our results from the time-dependent TGFβ1 inhibition assays (Figure 2a,b,c) and RT-qPCR analysis for NFATc1 from PBMs cultured for 48 h (Figure 4a), both of which suggest that TGFβ1 inhibits RANKL-stimulated human osteoclastogenesis at the early stage of osteoclast precursors. Further, we observed relatively increased osteoclast generation following RANKL and TGFβ1 treatment of PBMs from untreated seropositive RA patients, compared to healthy controls. Hence, PBMs from RA patients may exhibit low reactivity with TGFβ1. These findings indicate that TGFβ1 may represent a potential target for treating bone erosion in RA; in particular, TGFβ1 replacement therapy may improve the impaired bone homeostasis in RA.

## 4. Materials and Methods

### 4.1. Preparation of PBMs

This study was approved by the Ethical Committee for Epidemiology of Hiroshima University (approval number: E-668; approval date: 01/02/2017). Written informed consent was obtained from all blood donors. Blood samples were collected from twelve healthy donors (40–91 (60.3 ± 18.0) years old) and four RA patients (51–87 (71.3 ± 15.0, *p = 0.24*) years old), with an equal male–female ratio. PBMCs were obtained from the collected blood, using Lympholyte-H (CEDARLANE, Burlington, NC, USA) density gradient centrifugation. Highly purified PBMs were isolated from PBMCs, using MACS microbeads (Pan Monocyte Isolation Kit, Miltenyi Biotec GmbH, Bergisch Gladbach, Germany), according to the manufacturer’s instructions. In this system, more than 92% of the isolated cells were CD14-positive, as determined by flow cytometric analysis.

### 4.2. In Vitro Osteoclastogenesis

PBMs were cultured in a 96-well tissue culture plate, at a density of 5.0 × 10^4^ cells/well. Cultures were maintained in 200 μL of α-minimal essential medium (α-MEM; Wako Pure Chemical Industries, Ltd., Osaka, Japan) containing 10% fetal bovine serum (FBS; Wako Pure Chemical Industries) and treated with 50 ng/mL of M-CSF (Wako Pure Chemical Industries), 100 ng/mL of RANKL (Wako Pure Chemical Industries), or both, for 5–6 days, in the presence of 0–100 ng/mL of TGFβ1 (Wako Pure Chemical Industries). We used 100 ng/mL of TGFβ1 mainly in this study due to the following three reasons. First, one previous study reported that the serum levels of TGFβ1 in both healthy controls and RA patients were 100–200 ng/mL, while another study reported serum levels of TGFβ1 in healthy controls and RA patients to be 30–40 ng/mL and 40–50 ng/mL, respectively, so we regarded 1–100 ng/mL TGFβ1 as appropriate physiological in vivo levels for humans [61,62]. Second, considering the results of bone resorption assay (Figure 2e), we think 100 ng/mL of TGFβ1 shows the sufficient inhibitory effect on osteoclastogenesis in any assay systems. Third, the suppressive effect of TGFβ1 varies among different individuals. To cover the difference, enough concentration of TGFβ1 was required. In one experiment, PBMs were pretreated with 10 μg/mL of anti-TGFBRII antibody (R&D Systems, Minneapolis, MN, USA) diluted with α-MEM containing 10% FBS, for 5 min. Every 3 days, half of the culture medium was replaced with fresh medium containing the appropriate cytokines and chemicals.

### 4.3. TGFβ1 Cytotoxicity

PBMs were cultured with increasing concentrations (0–100 ng/mL) of TGFβ1, for 4 h. The supernatants were then collected and analyzed with a lactate dehydrogenase (LDH) release cytotoxicity detection kit (Sigma-Aldrich Co., St Louis, MO, USA) and presented as the optical density (OD) at 490 nm, according to the manufacturer’s instructions.

### 4.4. Cell Proliferation Assay

PBMs were cultured with M-CSF (50 ng/mL) and RANKL (100 ng/mL), in the presence or absence of TGFβ1 (100 ng/mL), for four days. Cell proliferation was then analyzed with a cell proliferation ELISA kit, bromodeoxyuridine (BrdU) colorimetric assay (Sigma-Aldrich Co.) and presented as the optical density (OD) at 450 nm, according to the manufacturer’s instructions.

### 4.5. Preparation of PBMs from Untreated Seropositive Patients with RA and In Vitro Osteoclastogenesis

Highly purified PBMs were obtained from four RA patients who complied with the American Rheumatism Association’s 1987 revised criteria for the classification of rheumatoid arthritis [63]. They were all seropositive (positive for anti-cyclic citrullinated peptide antibody) patients who were never treated with disease-modifying antirheumatic drugs but were treated with non-steroidal anti-inflammatory drugs. The effect of TGFβ1 on RANKL-stimulated osteoclast formation in PBMs obtained from healthy donors and RA patients was evaluated with TRAP staining.

### 4.6. TRAP Staining

Five to six days after baseline, the cells were fixed and stained for TRAP, using an acid phosphatase, leukocyte (TRAP) staining kit (Sigma-Aldrich Co.), according to the manufacturer’s instructions. Cells that were stained dark red were considered to be differentiated osteoclast-like cells. The TRAP-positive MNCs in the 96-well plates were counted, using a light microscope (EVOS XL Core Cell Imaging System; Thermo Fisher Scientific, Fremont, CA, USA). To evaluate the dose-dependent effect of TGFβ1 on RANKL-induced osteoclastogenesis in human PBMs, PBMs were cultured with 50 ng/mL of M-CSF and/or 100 ng/mL of RANKL in the presence of increasing concentrations (0–100 ng/mL) of TGFβ1 for 5–6 days. To evaluate the time-dependent effect of TGFβ1, TGFβ1 (100 ng/mL) was added to the PBM culture system at four time points (0, 24, 48, and 72 h after baseline) and was maintained until the end of incubation. The number of TRAP-positive MNCs at the time of TGFβ1 addition was subtracted from those at the end of the culture period to normalize the number of MNCs and evaluate the net effect of TGFβ1 on the number of MNCs. Next, to evaluate the differentiation stage-dependent effect of TGFβ1, TGFβ1 (100 ng/mL) was added to the PBM culture system at different stages of differentiation for 24 h.

### 4.7. In Vitro Assays for Osteoclast Resorption

To measure bone-resorptive function, PBMs were plated at a density of 5 × 10^4^ cells/well on an Osteo assay surface plate (Corning, New York, NY, USA) and cultured with 50 ng/mL of M-CSF and/or 100 ng/mL of RANKL in the presence of increasing concentrations (0–100 ng/mL) of TGFβ1 for 7–12 days, followed by cell lysis and silver nitrate staining. The Osteo assay surface plate has an inorganic crystalline calcium phosphate coating that mimics living bone material. The osteoclasts were lysed by using osmotic shock and stained with 5% silver nitrate (Sigma-Aldrich Co.) in PBS, to quantify the resorption area, using an EVOS microscope with ImageJ software. To evaluate the effect of TGFβ1 on mature osteoclast activity, PBMs were cultured with M-CSF (50 ng/mL) and/or RANKL (100 ng/mL), with or without TGFβ1 (100 ng/mL), 0 or 144 h after baseline, and maintained until the end of incubation. Bone-resorptive function was analyzed ten days after baseline.

### 4.8. Flow Cytometric Analysis

Both CD14 and CD11b expression on the surface of PBMs and osteoclast precursors from three different healthy donors were analyzed by flow cytometry. Human PBMs were cultured with M-CSF (50 ng/mL) and RANKL (100 ng/mL), with or without TGFβ1 (100 ng/mL), for 120 h, and the plastic-dish-adherent cells were harvested as osteoclast precursors, using Accutase (Innovative Cell Technologies, San Diego, CA, USA) treatment, prior to flow cytometric analysis. The PBMs and osteoclast precursors were first incubated with anti-Fc antibody (BioLegend, San Diego, CA, USA), at 20–25 °C, for 15 min, to block the Fc receptors. Subsequently, the cells were washed twice with PBS supplemented with 2 mM of EDTA and 0.5% (*w*/*v*) BSA (Wako Pure Chemical Industries), and incubated with fluorescein isothiocyanate (FITC)-conjugated anti-human CD14 antibodies (clone M5E2, BioLegend) and APC-conjugated anti-human CD11b antibodies (clone ICRF44, BioLegend), for 30 min, at 4 °C. FITC- and APC-conjugated mouse IgG (BioLegend) were similarly incubated as isotype controls. The cells were then analyzed by using a CytoFLEX flow cytometer (Beckman coulter, Brea, CA, USA), and the data were collected and analyzed by using the Kaluza software (Beckman coulter).

### 4.9. Real-Time Reverse Transcription PCR (RT-qPCR) Analysis

Cells were lysed, and total RNA was isolated by using a NucleoSpin RNA kit (Takara-Bio, Shiga, Japan). The total RNA (200 ng) was then reverse-transcribed to cDNA, using a PrimeScript™ RT reagent kit with gDNA Eraser (Takara-Bio). RT-qPCR was then performed, using TB Green *Premix Ex Taq* II (Tli RNaseH Plus) (Takara-Bio) with the CFX Connect real-time PCR detection system (BioRad Laboratories, Inc., Hercules, CA, USA). PCR amplification was performed with 40 cycles at 95 °C for 5 s and 60 °C for 10 s. The primers used in this study are listed in Table 1. Gene expression was normalized to *ACTB* for each sample.

### 4.10. Western Blotting

PBMs cultured with M-CSF and both or either of RANKL and TGFβ1 for 120 h were washed with PBS, and total protein was extracted by using the radio-immunoprecipitation assay (RIPA) buffer (50 mM Tris-HCl pH 8.0, 150 mM sodium chloride, 0.5% (*w/v*) sodium deoxycholate, 0.1% (*w*/*v*) sodium dodecyl sulfate (SDS), 1.0% (*w*/*v*) NP-40 substitute; Wako Pure Chemical Industries). Total cell lysates were clarified by centrifugation at 12,000 rpm, for 10 min, at 4 °C. Equal protein levels were determined and adjusted by the Pierce BCA protein assay kit (Thermo Scientific, Waltham, MA, USA), according to the manufacturer’s instructions. Samples were subjected to 10% SDS-polyacrylamide gel (Wako Pure Chemical Industries) electrophoresis and transferred to polyvinylidene fluoride (PVDF) membranes (Novex; Thermo Fisher Scientific). Subsequently, the membranes were blocked with TBS-T (25 mM Tris-HCl pH 7.4, 0.1% (*w*/*v*) Tween-20, 137 mM NaCl, 2.68 mM KCl; Wako Pure Chemical Industries) containing 4% skimmed milk powder (Wako Pure Chemical Industries) for a few hours, at 20–25 °C. Then, the membranes were incubated overnight at 4 °C with primary antibodies against NFATc1 (1:500; mouse monoclonal; clone 7A6; BioLegend), Cathepsin K (1:250; rabbit polyclonal; ab19027; Abcam, Cambridge, UK), and β-actin (1:500; rabbit polyclonal; clone Poly6221; BioLegend); the antibodies were diluted with TBS-T containing 4% skimmed milk powder. The membranes were then incubated with horse radish peroxidase-conjugated secondary antibodies (1:1000; Cell Signaling Technology, Tokyo, Japan), for 1 h, at 20–25 °C. The membranes were washed with TBS-T several times between blocking and incubation procedures. Protein signals were developed by using an enhanced chemiluminescence reagent (ImmunoStar LD; Wako Pure Chemical Industries) and the ImageQuant LAS 500 system (GE Healthcare, Chicago, IL, USA), according to the manufacturer’s instructions.

### 4.11. Plasmid Construction

For the luciferase assay, pGL4-NATc1p plasmid was constructed by inserting custom human NFATc1 promoter region (from −624 to +105 bp relative to the TSS) (GenScript, Piscataway, NJ, USA) into the pGL4.16 vector (Promega, Madison, WI, USA). For overexpression experiments, pCMV-TGFβRII was obtained from M. Inui (Meiji University), and pCMV3-myc c-Fos was purchased from Sino Biological Inc (Wayne, PA, USA). Then, pcDNA3-myc p65 was constructed. Briefly, full-length cDNA of RelA (p65) (*RELA*; NM_021975.4) was harvested by PCR and inserted into the pcDNA3 vector (Invitrogen, Carlsbad, CA, USA).

### 4.12. Luciferase Assay

HEK293T cells (1.0 × 10^5^ cells/well in a 12-well plate) were transfected with the reporter plasmid (pGL4-NFATc1p), with or without pcDNA3-myc p65, pCMV3-myc c-Fos, pCMV5-TGFβRII, pRL-SV40, and a mock plasmid (pcDNA3 vector) for 4–6 h, using Lipofectamine 3000 (Invitrogen) and FBS-free Dulbecco’s modified Eagle’s medium (DMEM; Wako Pure Chemical Industries). Subsequently, the cells were rinsed with PBS and incubated with DMEM containing 1% FBS, for 24–36 h. After transfection, recombinant TGFβ1 was added to the culture for 4–6 h prior to lysis. Following incubation, cells were lysed and analyzed with luciferase assay reagent (Promega), or the dual-luciferase reporter assay system (Promega), in a 96-well plate. Readings of the luciferase activity were obtained by using the Tristar LB941 multiplate reader (Berthold Technology, Bad Wildbad, Germany).

### 4.13. Immunofluorescence Analysis

PBMs were cultured in a 6-well tissue culture plate, with cover slips in the center of each well, at a density of 1.0 × 10^6^ cells/well. Cultures were maintained in 1500 μL of α-MEM supplemented with 10% FBS and treated with M-CSF (50 ng/mL), in the presence or absence of TGFβ1, for 4 h, followed by RANKL (100 ng/mL) addition to each well for 30 min. Cells were incubated in TBS and TBS-T for 5 min, twice. PBMs were blocked with 5% Blocking One P (Nacalai Tesque, Kyoto, Japan) diluted with TBS for 1 h at 20–25 °C. Cells were then incubated overnight, at 4 °C, with primary antibodies against p65 (1:150; rabbit polyclonal; D14E12; Cell Signaling Technology), followed by incubation with Alexa Fluor 488-conjugated anti-rabbit IgG antibodies (1:1,000; host: Goat; Cell Signaling Technology) for 1 h, at 20–25 °C. The cells were washed with TBS several times between blocking and incubation procedures. Subsequently, cells on coverslips were enclosed on slide glasses with VECTASHIELD mounting medium with DAPI (Funakoshi, Tokyo, Japan). Then, p65 nuclear translocation images were obtained, using a digital microscope VHX-7000 (KEYENCE, Osaka, Japan).

### 4.14. Statistical Analysis

All experiments were performed in triplicate or quadruplicate. All graphs show one representative experiment, and the data represent mean ± standard deviation values. All statistical analyses were performed by using the Student’s *t-*test (paired, 2-tailed). A *p*-value < 0.05 was considered to be statistically significant. Data were processed and analyzed by using the GraphPad Prism 8 software (Graph Pad Software Inc., La Jolla, CA, USA).

## 5. Conclusions

Our study suggests that TGFβ1 suppresses osteoclast generation in humans via downregulation of NFATc1, by blocking nuclear translocation of p65. It provides a foundation for elucidating the precise mechanisms underlying the role of TGFβ1 in osteoclastogenesis and for developing novel therapeutic strategies for RA.

## Figures and Tables

**Table 1:** Real-time reverse-transcription PCR primer sequences for genes used in this study.

Gene	Sense	Anti-Sense
*NFATC1*	5′-GCATCACAGGGAAGACCGTGTC-3′	5′-GAAGTTCAATGTCGGAGTTTCTGAG-3′
*CTSK*	5′-AGTTTTACAGCAAAGGTGTG-3′	5′-CTTGTTTCCCTTCTGGATTC-3′
*P65*	5′-GAGACATCCTTCCGCAAACT-3′	5′-TCCTTCCTGCCCATAATCA-3′
*C-FOS*	5′-CAGTTATCTCCAGAAGAAGAAG-3′	5′-CTTCTAGTTGGTCTGTCTCC-3′
*ACTB*	5′-GACGACATGGAGAAAATCTG-3′	5′-ATGATCTGGGTCATCTTCTC-3′

*NFATC1* = nuclear factor of activated T cells, cytoplasmic 1; *CTSK* = cathepsin K; *ACTB* = beta actin.

## Supplementary Materials

The following are available online at https://www.mdpi.com/1422-0067/21/3/800/s1. Figure S1: Cytotoxicity of TGFβ1 on human peripheral blood monocytes. Figure S2: The stimulatory effect of TGFβ1 on cell proliferation during osteoclastogenesis. Figure S3: The effect of TGFβ1 on mature osteoclast activity. Figure S4: Differentiation stage-dependent inhibition of osteoclastogenesis and bone resorption by TGFβ1 in humans. Figure S5: Dual luciferase assay revealed the inhibitory effect of TGFβ1 on NFATc1 promoter activity.

## Abbreviations

ACTB	beta actin
CTSK	cathepsin K
HC	healthy control
M-CSF	macrophage-colony stimulating factor
MFI	mean fluorescence intensity
MNC	multinucleated cell
NF-κB	nuclear factor kappa-B
NFATc1	nuclear factor of activated T cells, cytoplasmic 1
PCR	polymerase chain reaction
PBM	peripheral blood monocyte
PBMC	peripheral blood mononuclear cell
RA	rheumatoid arthritis
RANKL	receptor activator of nuclear factor kappa-B ligand
TGFβ1	transforming growth factor beta1
TGFBRII	TGFβ1 receptor II
TRAP	tartrate-resistant acid phosphatase
