# Supplementary material for: TGFβ1 Regulates Human RANKL-Induced Osteoclastogenesis via Suppression of NFATc1 Expression
Source: Int J Mol Sci. 2020 Jan 25;21(3):800. doi: 10.3390/ijms21030800 (PMC7038124; doi:10.3390/ijms21030800)
Supplement: Supplementary file 1 [file ijms-21-00800-s001.pdf]

Supplementaly Figures (Figure S1-S5)

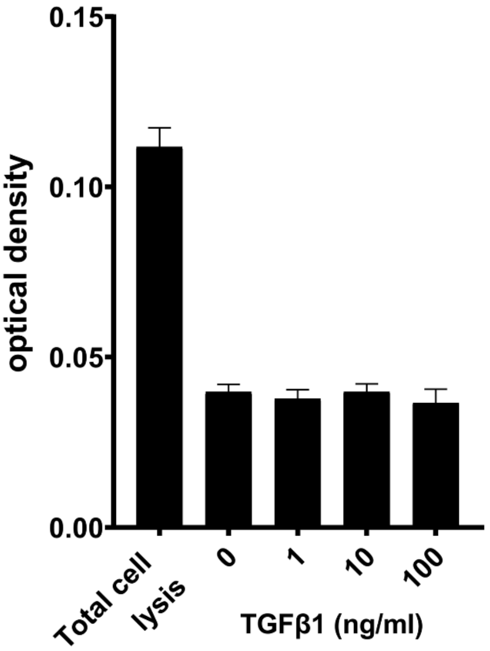

Figure S1. Cytotoxicity of TGFβ1 on human peripheral blood monocytes (PBMs). Human PBMs were cultured with increasing concentrations (0-100 ng/mL) of TGFβ1 for 4 h. Cytotoxicity activity was measured using the relative levels of lactate dehydrogenase (LDH), and presented as the optical density (OD) at 490 nm. The data are presented as the mean ± standard deviation.

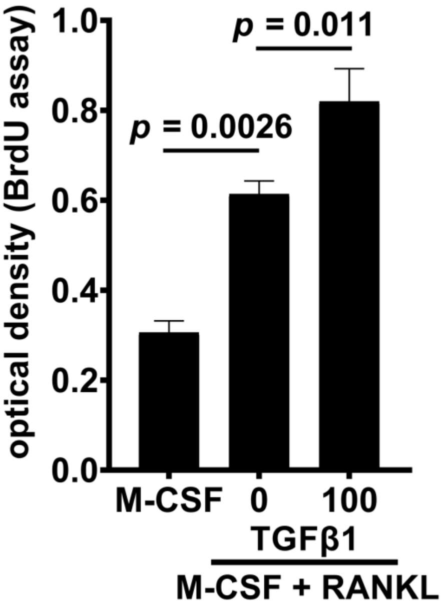

Figure S2. The stimulatory effect of TGFβ1 on cell proliferation during osteoclastogenesis. PBMs were cultured with M-CSF (50 ng/ml) and RANKL (100 ng/ml) in the presence or absence of TGFβ1 (100 ng/ml) for 4 days. The cell proliferation activity of cultured cells was determined by a bromodeoxyuridine (BrdU) colorimetric assay, and presented as the optical density (OD) at 450 nm. The data are presented as the mean ± standard deviation.

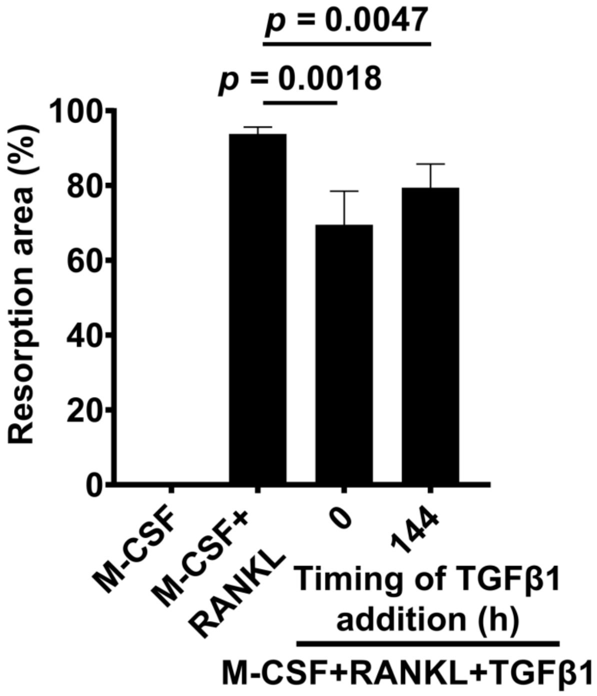

Figure S3. The effect of TGFβ1 on mature osteoclast activity. PBMs were cultured with M-CSF (50 ng/ml) and/or RANKL (100 ng/ml) and TGFβ1 (100 ng/mL) was added to the culture system at 0 or 144 h after baseline, followed by cell lysis and silver nitrate staining 10 days after baseline. TGFβ1 inhibited RANKL-mediated bone resorption activity of mature osteoclasts. During the culture, half the culture medium was replaced with fresh medium containing cytokines and TGFβ1 every 3 days. The data are presented as the mean ± standard deviation.

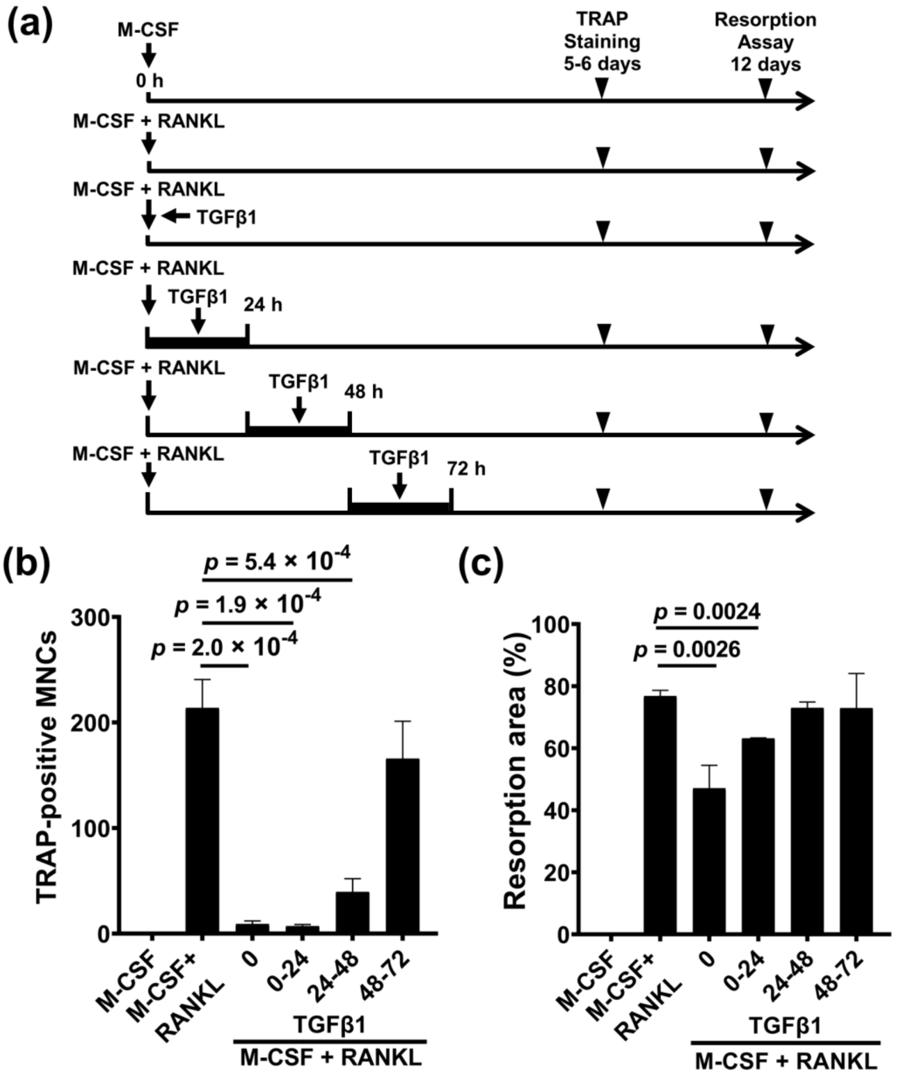

Figure S4. Differentiation stage-dependent inhibition of osteoclastogenesis and bone resorption by TGFβ1 in humans. (a) TGFβ1 (100 ng/mL) was added to the peripheral blood monocyte (PBM) culture system at the different stages of differentiation for 24 h. (b) The cells were cultured for 5-6 days and then analyzed by tartrate-resistant acid phosphatase (TRAP) staining. Addition of TGFβ1 throughout the culture period, between 0 and 24 h and between 24 and 48 h resulted in significant inhibition of RANKL-induced osteoclastogenesis. (c) Bone resorption activity was analyzed using Osteo assay plates. PBMs were cultured on Osteo assay surface 96-well plates for 12 days, followed by cell lysis and silver nitrate staining. TGFβ1 throughout the culture period and between 0 and 24 h significantly inhibited RANKL-mediated bone resorption activity. During the culture, half the culture medium was replaced with fresh medium containing cytokines and TGFβ1 every 3 days. The data are presented as the mean ± standard deviation.

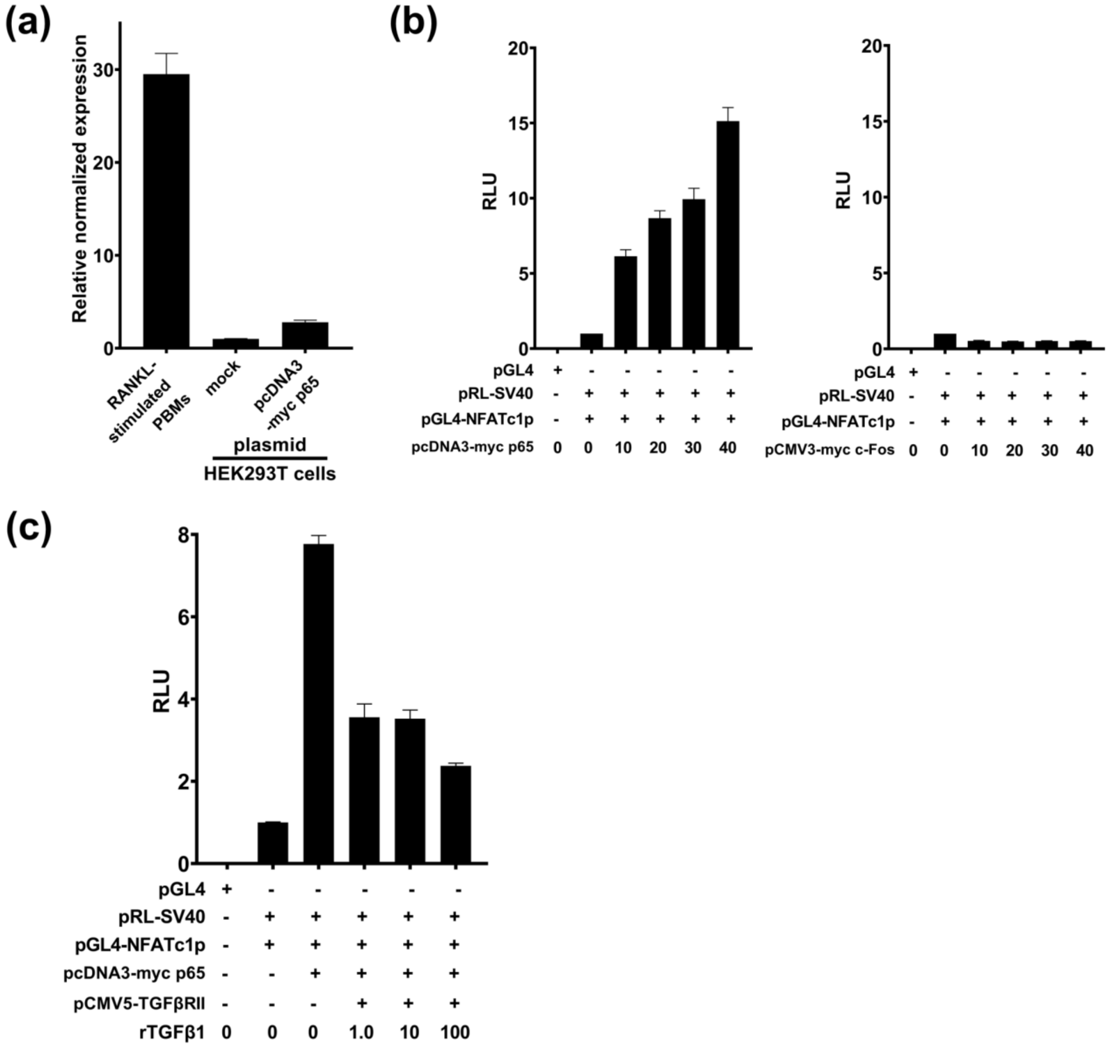

Figure S5. Dual luciferase assay revealed the inhibitory effect of TGFβ1 on NFATc1 promoter activity. (a) Gene expression of *NFATC1* was measured by real-time reverse transcription polymerase chain reaction (RT-qPCR). Total RNA from PBMs cultured with M-CSF (50 ng/ml) and RANKL (100 ng/ml) for 48 h was harvested. Human embryonic kidney (HEK) 293T cells (1.0×10^5 cells/well in a 12-well plate) were transfected with the indicated plasmids [pcDNA3-myc p65 or mock plasmid (pcDNA3 vector)]. After transfection, total RNA from HEK293T cells was cultured for 24 h prior to lysis. The data were normalized to *ACTB* expression and are presented as the mean ± standard deviation ($n = 3$). (b-c) Dual luciferase assay using pRL (Renilla) plasmid. (b) HEK 293T cells were transfected with the indicated plasmids [pGL4-basic plasmid, 200 ng; reporter plasmid (pGL4-NFATc1p), 200 ng; pcDNA3-myc p65, 0–40 ng; pCMV3-myc c-Fos, 0–40 ng; pRL-SV40 plasmid; and mock plasmid (pcDNA3 vector)]. (c) HEK293T cells were transfected with the indicated plasmids described above. After transfection, recombinant TGFβ1 (rTGFβ1; 0–100 ng/mL) were added to the culture for 4–6 h prior to lysis. The data are presented as the mean ± standard deviation.
